# Lipid metabolism analysis in liver of different chicken genotypes and impact on nutritionally relevant polyunsaturated fatty acids of meat

**DOI:** 10.1038/s41598-022-05986-2

**Published:** 2022-02-03

**Authors:** Alice Cartoni Mancinelli, Alessandra Di Veroli, Simona Mattioli, Gabriele Cruciani, Alessandro Dal Bosco, Cesare Castellini

**Affiliations:** 1 Department of Agricultural, Environmental and Food Science, University of Perugia, Borgo XX Giugno, 74, 06123 Perugia, Italy; 2 Department of Chemistry, Biology and Biotechnology, University of Perugia, Via Elce di Sotto, 8, 06123 Perugia, Italy

**Keywords:** Biochemistry, Systems biology

## Abstract

Humans and mammalian species are unable to synthesize significant amounts of polyunsaturated fatty acids (PUFA), which therefore must be introduced with the diet. In birds, lipogenesis takes place primarily in the liver, whereas adipose tissue serves as the storage site for triacylglycerols (TG, composed by 80–85% esterified fatty acids). However, both the nature (unsaturation level, n-3, or n-6 series) and the allocation (such as constituents of complexed lipids) of PUFA are very important to evaluate their function in lipid metabolism. The objective of the present investigation was to study the liver lipid metabolism, with particular attention to non-esterified fatty acids (NEFA), TG, phospholipids (PL), FADS2 gene expression, and Δ6-desaturase activity of three chicken genotypes, Leghorn (Leg), Ross 308 (Ross), and their crossbreed (LxR), by LC/MS analysis. The concentration of single fatty acids in muscle was quantified by GC-FID. The results showed that the Ross has a lipid metabolism related mainly to storage and structural roles, exhibiting higher levels of TG, phosphatidylethanolamine (PE) and phosphatidylcholine (PC) that are largely unsaturated. Meanwhile Leg showed a relevant amount of n-3 NEFA characterized by a higher phosphatidylserine (PS) unsaturation level, FADS2 gene expression and enzyme activity. The LxR seem to have a moderate trend: n-6 and n-3 NEFA showed intermediate values compared with that of the Ross and Leg and the TG trend was similar to that of the Ross, while PE and PC were largely unsaturated (mainly 6 and 7 UNS most of the metabolic energy for storage fatty acids in their tissues (TG) whereas, the Leg birds were characterized by different lipid metabolism showing in their liver a higher content of n-3 NEFA and higher unsaturation level in PS. Furthers details are needed to better attribute the lipid energy to the different metabolic portion.

## Introduction

The human intake of polyunsaturated fatty acids (PUFA) in Western countries is unbalanced, with a predominance of n-6 with respect to the n-3 series, due to the current consumption of foods with high n-6 PUFA content^1^. Accordingly, in the last 100 years, the n-6/n-3 ratio of common foods reached a value of 20:1 when the recommendation dietary intake was equal or less than 4:1^2,3^, with a consequent negative effect on human health, i.e., increases in cardiovascular, neurodevelopmental and psychiatric disorders^4–6^.

Humans and mammalian species are unable to synthesize significant amounts of PUFA which therefore must necessarily be introduced with the diet^7^. The PUFA may be ingested as precursors: α-linolenic acid (ALA, C18:3n-3) for the n-3 series and linoleic acid (LA, C18:2n-6) for the n-6 series, which are essential fatty acids, or as derivatives (long chain PUFA, LC PUFA), with more than 20 carbon atoms.

The health benefits of LC PUFA and consequently the production of foods rich in n-3 PUFA is an important goal for the food industry. Indeed, fish which are currently the major source of n-3 LC PUFA, cannot meet through fishing and aquaculture the worldwide demand for these bioactive compounds^4,5^. Thus, one of the main strategies to balance the n-6/n-3 ratio in the human diet should be to improve the n-3 PUFA content in animal products. In this context, it should be underlined the close relation between the fatty acid (FA) profile of feed and products in poultry meat^8^, which, in turn, is the most produced meat in the world (37.27% of total production)^9^.

Specifically, chicken can convert ALA into eicosapentaenoic (EPA, C20:5n-3), docosapentaenoic (DPA, C22:5n-3), and docosahexaenoic (DHA, C22:6n-3) acids, and the same enzyme convert LA, into arachidonic acid (AA, C20:4n-6)^10,11^. Fatty acids with carbon skeletons > 20 such as EPA, DPA, DHA and AA comprise LC PUFA and are generated from fatty acids with < 20 carbons by specific elongases and desaturases^7^. The Δ-6 desaturases (Δ6d), is considerate the most limiting step because it acts twice in the biosynthetic process^12^.

The scientific literature reports that there are many factors affecting PUFA metabolism and the substrate preference of the Δ6d enzyme for LA or ALA, such as species^13,14^, genotype, age^15,16^, sex^17^, diet^8,18,19^ and rearing system^20–22^. High-performance genotypes use most of the dietary energy for muscle development rather than for other metabolic processes (kinetics, reproduction, immune response, thermotolerance, etc.)^23,24^. Thus, the growth rate largely affects the metabolic activity in fast-growing chickens in terms of PUFA accumulation^25^ and in modification of metabolic pathways^26^.

Considering that lipogenesis takes place primarily in the liver of chickens, the aim of the present investigation was to compare the lipid metabolism in three genotypes of poultry whose growth rates and muscular development differed. The liver of Leghorn (slow-growing genotype, SG), Ross 308 (fast-growing genotype, FG) and their crossbreed (Leghorn x Ross 308 as a medium growing genotype, MG) was analyzed by a lipidomic approach for identify the main functional lipid classes (non-esterified fatty acids—NEFA, triacyclglycerols—TG and phospholipids – PL). Fatty Acid Desaturase 2 (FADS2) gene expression and Δ6d activity of liver were evaluated. Finally, to assess the effect of liver metabolism on the characteristics of meat, the FA profile of muscle was also determined with a GC-FID.

## Results

### Fatty acid profile of NEFA

**Figure 1:**
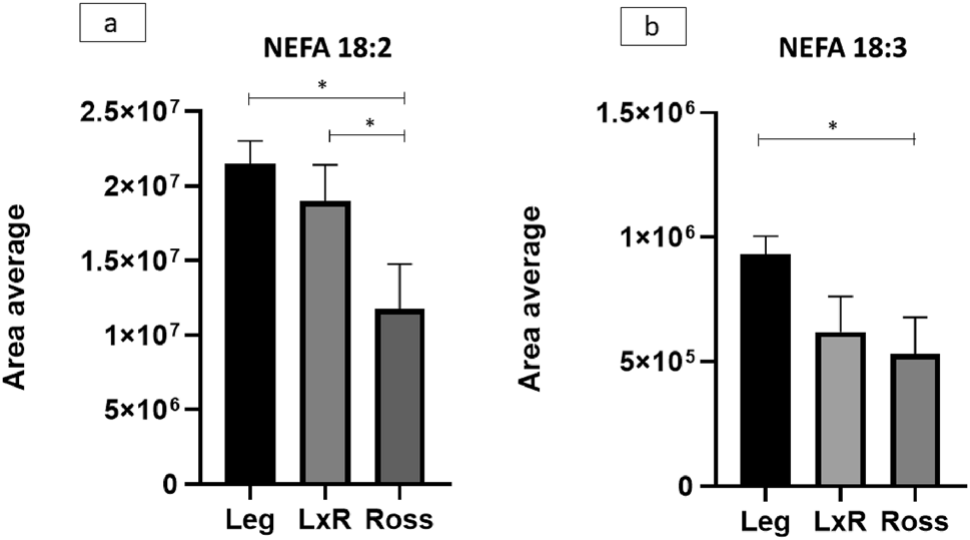
n-6 and n-3 non-esterified fatty acids (NEFA) of chicken liver: (**a**) 18:2 (linoleic acid, LA; precursor of n-6 LC PUFA); (**b**) 18:3 (α-linolenic acid, ALA; precursor of n-3 LC PUFA). Leg: Leghorn genotype; LxR: crossbreed, Ross: Ross 208 genotype. *P < 0.05 (post hoc Tukey’s test).

**Figure 2:**
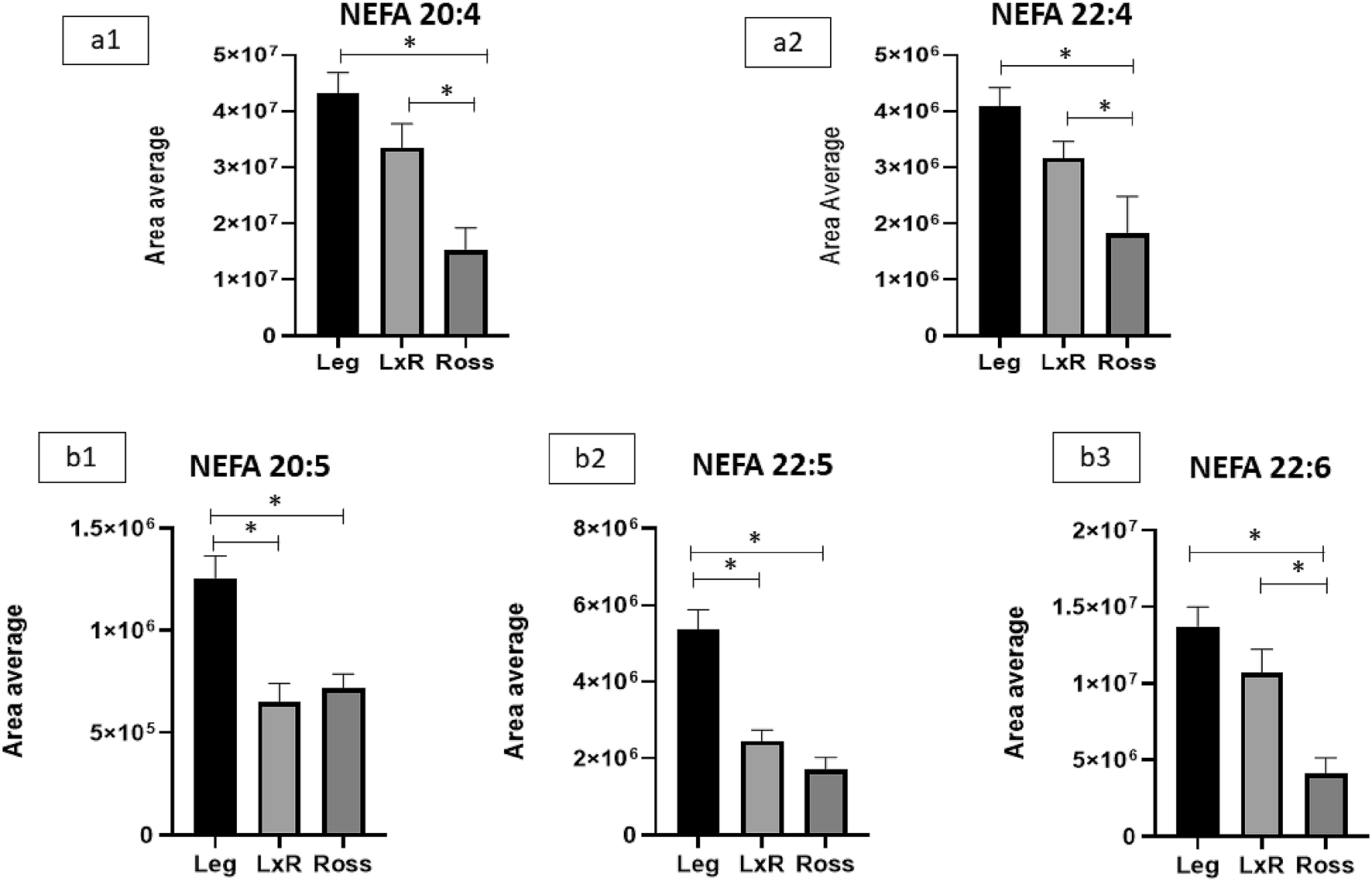
n-6 and n-3 NEFA of chicken liver (**a**) LC PUFA from the n-6 series: NEFA 20:4 (arachidonic acid, ARA, (**a1**); NEFA 22:4 (adrenic acid, AdA; (**a2**). (**b**) LC PUFA from the n-3 series: NEFA 20:5 (eicosapentaenoic acid, EPA; NEFA 22:5 (docosapentaenoic acid, DPA; **b2**); FA 22:6 (docosaexaenoic acid, DHA; **b3**). Leg: Leghorn genotype; LxR: crossbreed, Ross: Ross 208 genotype. *P < 0.05 (post hoc Tukey’s test).

In Figs. 1 and 2, the NEFA (precursors: ALA and LA and derivatives: LC PUFA) of the liver are reported. The concentrations of NEFA in both the n-6 and n-3 series exhibited the same trend among the genotypes.

**Figure 3:**
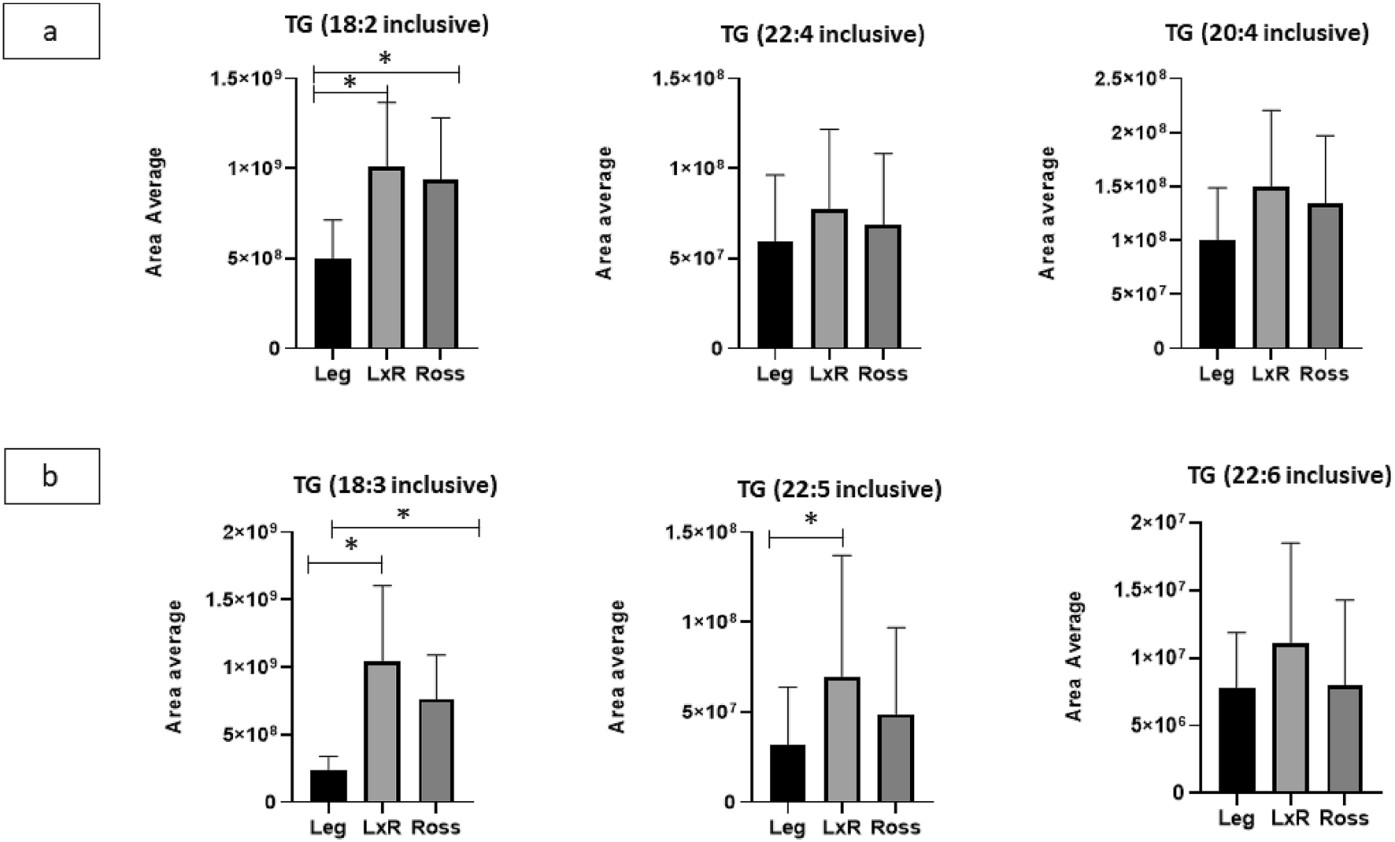
n-6 and n-3 triacylglycerols (TG) of chicken liver: TG, which included specific PUFA (**a**) from n-6 (18:2, LA; 20:4, ARA; 22:4, AdA) and (**b**) from n-3 series (18:3, ALA; 22:5, DPA; 22:6, DHA). Leg: Leghorn genotype; LxR: crossbreed, Ross: Ross 208 genotype. *P < 0.05 (post hoc Tukey’s test).

**Figure 4:**
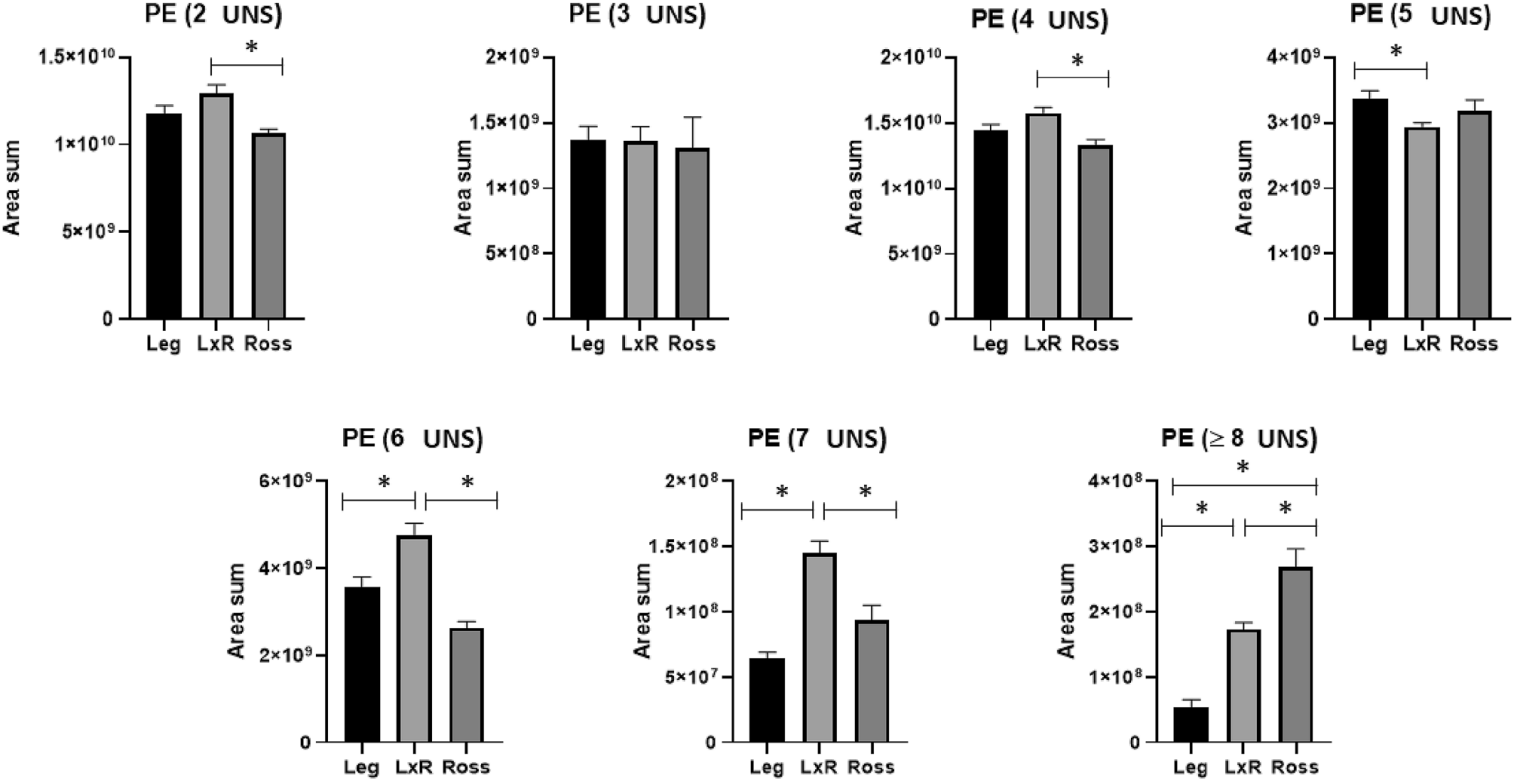
Phosphatidylethanolamine (PE) with different unsaturation levels (from 2 to ≥ 8 UNS). Leg: Leghorn genotype; LxR: crossbreed, Ross: Ross 208 genotype. *P < 0.05 (post hoc Tukey’s test).

**Figure 5:**
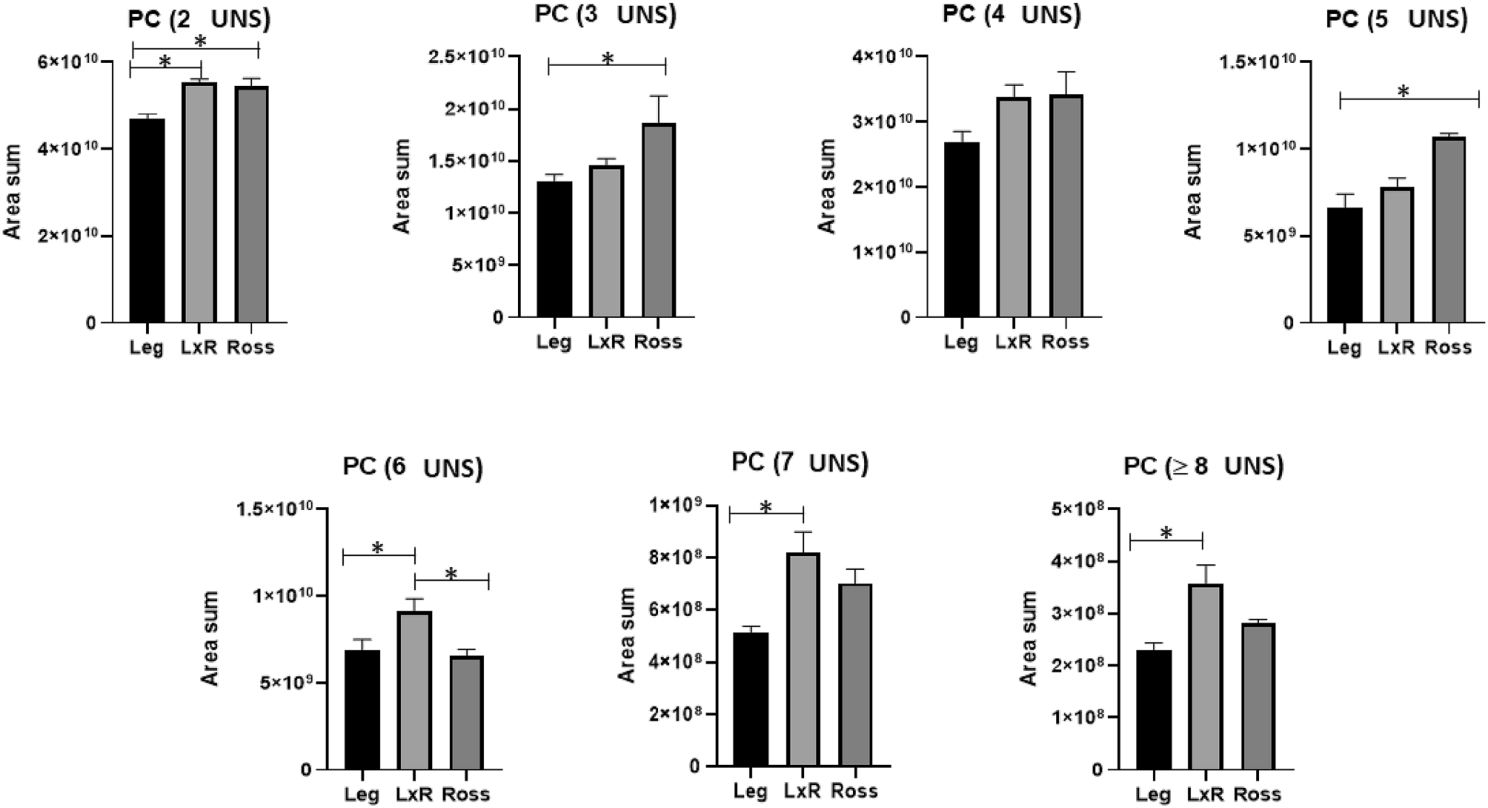
Phosphatidylcholine (PC) with different unsaturation levels (from 2 to ≥ 8 UNS). Leg: Leghorn genotype; LxR: crossbreed, Ross: Ross 208 genotype. *P < 0.05 (post hoc Tukey’s test).

**Figure 6:**
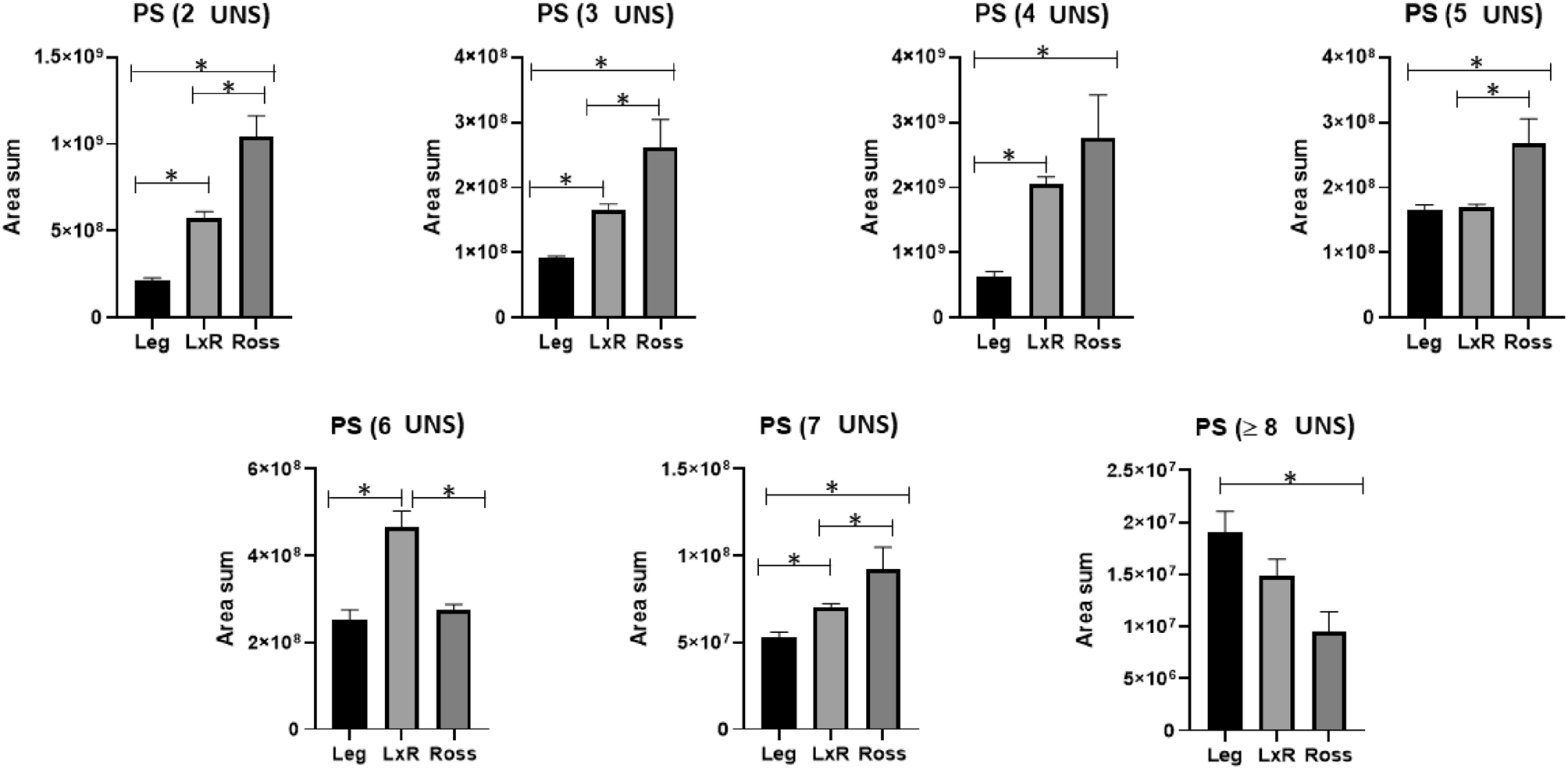
Phosphatidylserine (PS) with different unsaturation levels (from 2 to ≥ 8 UNS). Leg: Leghorn genotype; LxR: crossbreed, Ross: Ross 208 genotype. *P < 0.05 (post hoc Tukey’s test).

Precursors (LA, Fig. 1a) and n-6 NEFA derivatives (arachidonic-ARA and adrenic-AdA acids; Fig. 2a1 and a2) showed similar trends, with higher values for the Leg genotype, lower values for the Ross genotype and intermediate values for the LxR (P ≤ 0.05). Additionally, the n-3 NEFA precursors (ALA; Fig. 1b) and derivatives exhibited the same trend (Fig. 2b1-b3). The differences between LxR and Ross genotypes were significant, except for n-3 NEFA: ALA, EPA and DPA.

### Fatty acid profile of TG

In Fig. 3, some traits of the FAs chains included in the TG of chicken liver are shown.

The TG of the LxR and Ross genotypes were not significantly different in the precursors of the n-6 (Fig. 3a) and n-3 (Fig. 3b) series, respectively. However, the LxR and Ross genotypes exhibited higher levels than the Leg genotype for both TG precursors inclusive (LA and ALA), while with TG 22:5 inclusive, the difference was significant only with respect to the LxR genotype. No TG including EPA was found.

### Fatty acid profile of PL

Some features of the PL profile are reported in Figs. 4, 5, 6. The main classes of PL were phosphatidylethanolamine (PE, Fig. 4) and phosphatidylcholine (PC, Fig. 5). Phosphatidylserine (PC) represented minor amounts (Fig. 6).

The PE with less fewer than 5 unsaturated bonds (UNS) showed only partially significant differences among the genotypes; conversely, when UNS was ≥ 6, a distinct trend was shown. The LxR genotype showed a significantly higher value than the other genotypes; the pattern was LxR followed by Leg and Ross. Regarding PE with ≥ 8 UNS, the following pattern was found: Ross > LxR > Leg.

Phosphatidylcholine (PC) showed a different trend (Fig. 5). The Leg chickens showed a significantly lower amount of PC for all levels of unsaturation, except at 4 UNS, when compared with both other genotypes. However, the Ross genotype showed the highest value only at the lower unsaturation level (≤ 5 UNS); in contrast, in the crossbreed genotype, the value was higher at ≥ 6 UNS.

Figure 6 shows the phosphatidylserine (PS) unsaturation level in the different poultry genotypes. When the unsaturation level was lower than 4 and 7, a similar trend was found, with higher values for the Ross genotype, intermediate values for the LxR genotype and lower values for the Leg genotype. No differences between the Leg and LxR genotypes were found in PS with 5 UNS, while the Ross genotype remained the highest. A unique trend was recorded for PC with 6 UNS, showing a higher value for the LxR genotype; the PC with ≥ 8 UNS showed the tendency: Leg > LxR > Ross.

### FADS 2 gene expression and Δ6d activities

**Figure 7:**
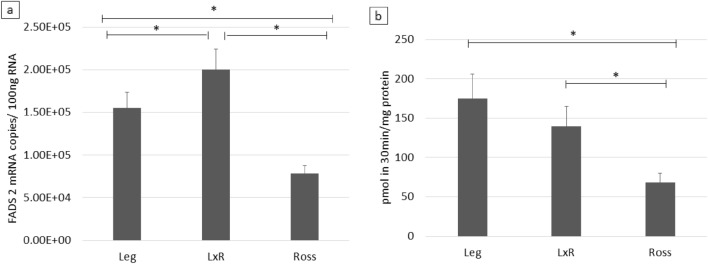
mRNA expression of FADS2 gene (mRNA copies/100 ng RNA; (**a**) and Δ6-desaturase activity (pmol in 30 min/mg of protein; (**b**) of liver. Leg: Leghorn genotype; LxR: crossbreed, Ross: Ross 208 genotype. *P < 0.05 (post hoc Tukey’s test).

In Fig. 7 the FADS2 gene expression and the Δ6d activity of chicken liver are reported. Data showed that FADS2 expression was upregulated in Leg and LxR respect to Ross. Similarly, in Leg and LxR the same (p > 0.05) enzyme expression was found, whereas Ross showed lower value.

### Muscle fatty acid profile

Table 1 reports the lipid content (g/100 g of muscle) and FA, expressed as quantitative (mg/100 g of fresh matter) and qualitative (% of total FA) data, for breast muscle.

**Table 1:** Total lipid content (g/100 g of fresh matter), n-3 and n-6 PUFA of breast muscle expressed as mg/100 g of fresh matter and % of total FA and estimated lipid enzyme activities.

	Leg	LxR	Ross	RMSE	P value
Lipids, g/100 g of f.m	1.47 A	2.50 B	3.51 C	0.256	0.026
**Fatty acids, mg/100 g of f.m**					
18:2n-6, LA	248.42 A	350.29 B	553.14 C	11.095	0.001
20:4n-6, AA	102.31 A	182.02 B	112.79 A	5.563	0.000
18:3n-3, ALA	29.52	32.89	37.32	2.657	0.098
20:5n-3, EPA	19.03 A	36.46 B	38.58 B	2.963	0.000
22:5n-3, DPA	11.61 a	25.33 b	15.79 a	2.661	0.010
22:6, DHA	9.28 a	14.91 c	7.46 a	1.961	0.008
n-6	350.74 A	532.32 B	665.94 C	11.229	0.000
n-3	69.44 A	109.61 C	99.16 B	4.301	0.000
n-6 LC PUFA	102.31 A	182.02 B	112.79 A	5.563	0.000
n-3 LC PUFA	39.92 a	76.72 b	61.84 b	3.970	0.032
**Fatty acids, % of total FA**					
18:2n-6, LA	20.35 C	16.82 A	18.97 B	0.594	0.001
20:4n-6, AA	8.38 B	8.77 B	3.87 A	0.720	0.000
18:3n-3, ALA	2.42 B	1.59 A	1.28 A	0.400	0.001
20:5n-3, EPA	1.56 AB	1.76 B	1.32 A	0.242	0.016
22:5n-3, DPA	0.95 ab	1.22 b	0.54 a	0.283	0.002
22:6, DHA	0.76 b	0.72 b	0.26 a	0.256	0.000
n-6	28.74 C	25.65 B	22.84 A	0.536	0.000
n-3	5.69 B	5.28 B	3.40 A	0.644	0.000
n-6 LC PUFA	8.39 B	8.77 B	3.87 A	0.264	0.001
n-3 LC PUFA	3.27 B	3.70 B	2.12 A	0.105	0.001
**Estimated enzyme activities**					
% n-6 in LCP	29.16	34.19	16.93	1.390	0.083
% n-3 in LCP	57.48	69.99	62.36	1.020	0.593
Estimated activity by n-6	0.41B	0.51B	0.20 A	0.161	0.000
Estimated activity by n-3	1.35 a	2.33 b	1.65 a	0.362	0.039

A, B on the same row differ for P < 0.001 (post hoc Tukey’s test).

a, b on the same row differ for P < 0.01 (post hoc Tukey’s test).

The results showed that the Ross genotype had a 2.3-fold higher quantity of total lipids than the Leg genotype (3.51 vs 1.47 g/100 g muscle, respectively). Furthermore, the FA were constituted mainly of LA with respect to what was recorded for the Leg and LxR genotypes (553.4 vs 248.42 and 350.29 mg/100 g fresh matter, respectively: Table 1). However, the LxR genotype exhibited a higher proportion of n-3 LC PUFA and AA than the Leg and Ross genotypes. Conversely, for the proportion of FA, the Leg and LxR genotypes showed higher percentages of both n-6 and n-3 LC PUFA than the Ross genotype (8.39 and 8.77 vs 3.87% for n-6 LC PUFA and 3.27 and 3.70 vs 2.12% for n-3 LC PUFA, respectively). In addition, Leg seemed to be more oriented toward n-6 LC PUFA production that Ross (29.16 vs 19.63% n-6 in LCP; P > 0.05), as also showed by the estimated n-6 activity value. On the contrary, the n-3 activity showed similar value in Ross and Leg.

## Discussion

In the present paper, we used an untargeted approach to obtain broad information about each genotype in order to assess the major differences among them in terms of liver metabolism (NEFA, TG and PL) and how the more nutritionally relevant FAs of breast muscle were affected by it.

The NEFA, TG and PL may originate from four different sources: i) de novo lipogenesis, ii) cytoplasmic triacylglycerol stores, iii) FA derived from TG or PL of lipoprotein remnants directly taken up by the liver, and iv) plasma NEFA released by adipose tissue^27^. In addition, the oxidation rate may also affect the lipid content: a higher oxidation, due to an increased energy demand, reduces the rate of esterification of NEFA in the liver^27^.

The relative importance of these different origins depends mainly on the animal species. In birds, de novo lipogenesis is very active with respect to the other process (i.e., oxidation, lipolysis), and the liver is the main site of lipid metabolism^28^. Because standard poultry diets have less than 10% of lipids, the liver plays a key role in providing lipids destined for the body tissues.

To our knowledge, these results demonstrated for the first time, that although SG chickens (Leghorn) had a high mobilization of n-3 “free PUFA” (NEFA) and TG, PL did not follow the same trend.

In the liver of the Leg, TG had a lower proportion of PUFA precursors (LA and ALA), whereas they are mainly located in the NEFA, perhaps these precursors travelling as free fatty acids were stored as metabolites (LC PUFA) in the fat or in the muscle. The FADS2 expression and Δ6d activity confirmed that in the Leg liver these traits were mostly expressed^25^ partly explaining the higher concentrations of n-3 LC PUFA (EPA, DPA, DHA) than in Ross. Accordingly, breast muscle of the Leg and mainly their crossbred showed the highest percentage of LC PUFA (both n-3 and n-6), although the estimated preference for n-3 was similar in Leg and Ross. These aspects should be better investigated to verify directly the preference of different genotype for n-3 or n-6 PUFA. It is probable that, part of n-3 LC PUFA produced by Leg liver, was used to supply the energy of these very active chickens thought the β-oxidation pathway^29^.

However, the higher lipid content of the Ross genotype (2.3-fold higher than the Leg genotype) resulted in a higher level of these FA in the muscle (Table 1). It is not simply to explain the trend of crossbreed but it can be supposed that LxR exhibited intermediate characteristics compared to their parents by showing a PUFA metabolism similar to that of Leg (up-regulation of FADS 2 gene and high Δ6d activity) with the ability to store a great quantity of LC PUFA in meat similar to the Ross.

Such features suggested that the de novo synthesis of LC PUFA is poor in Ross and most of the LC PUFA of the muscle come from dietary intake^25^ whereas in Leg and LxR mainly comes from its own elongation capability starting from dietary precursor.

In agreement with our results, Walzem et al.,^30^ demonstrated a strong difference in n-3 LC PUFA plasma of two bird lines selected respectively for growth rate.

It should be noted that the same experimental feed was used for the different chicken strains, having different dietary requirement, and this could marginally affect the Δ6d activity^31^. Moreover, it could be also argued that the maturity level of the three genotypes was different and that Leg and its crossbreed at the same age is less physiologically mature than Ross. This could partially affect some sexual hormones which are retained linked to LC PUFA synthesis^32^.

Dietary interventions alter lipogenesis and fat deposition in the chicken liver^33–35^. During periods of energy surplus, the FA synthesis occurs: lipid metabolism responds with higher TG synthesis by the liver with a higher VLDL export rate to adipose tissue^36^. In addition, the LC-PUFA typically provide one of two fatty acids on common PL that are critical structural components of membranes as well as cellular reservoirs of FAs used in signaling molecule production^36–38^. Phosphatidylcholine (PC) and phosphatidylethanolamine (PE) constitute the greatest two fractions of PL with high unsaturation level indices relative to other lipid reservoirs such as TG. A third fraction exists: PS is synthesized from preexisting PC or PE, and its FA composition depends strictly on the other PL compositions^39^. The FA composition of PC and PE, which is constituted mainly by LC PUFA^40^, is reflective of both dietary lipid composition and LC PUFA synthesis.

In our research, we found that PE showed a lower unsaturation level in the Leghorn^26^, in contrast to the results for TG and NEFA; however, a higher level (6–7 UNS) was also recorded in the crossbreed. Considering that PE is the key building block of membranes^41^, this trend suggests a SG metabolism less oriented to the LC PUFA storage; as a consequence, only a small amount of LC PUFA remained to be used as “structural FAs” (PL of the double layer of the membrane)^37^.

Thus, the abundance of largely unsaturated PE in Ross suggests the preference of this FG strain for structural/building metabolism. Indeed, PE is the main PL constituent of the membrane ^40^. Such a tendency may be connected with the ability of FG chickens to use a great part of metabolic energy to increase their body growth performance^30^ at the expense of other physiological functions (immune system, disease or environmental resistance, adaptability, etc.)^23^. Accordingly, Mohammadigheisar et al.^24^, demonstrated that the growth rate and the utilization efficiency of energy are closely linked in broilers.

Although, no specific composition in n-3 or n-6 FAs in PL can be assessed it is possible to suppose that the increased unsaturation level arose from ≥ 8 UNS PL, whose component FAs would be expected to contain 22:6, 18:2-PL, or 20:5,18:3-PL or 20:4, 20:4-PL as well as others that contain one or more LC-PUFA (Supplemental material Table S1). Hence, what was reported suggests that in the ≥ 8 UNS PL evaluations, more than one LC PUFA was included.

Considering the PC, the crossbreed, as already noticed for TG, showed a higher unsaturation level, confirming the higher capacity of this genotype to retain LC PUFA. Conversely, PS showed higher UNS levels in the Leg genotype; such results were not surprising considering that PS has a high ability to incorporate n-3 LC PUFA, particularly DHA, during its biosynthesis^41^. In agreement, Hamilton et al.^42^ demonstrated in rats that variations in membrane DHA composition have a profound influence on PS accumulation in neuronal tissues, with a strong reduction of such molecules. Similarly, the higher quantity of liver DHA and EPA, which results in SG chickens rather than FG, could modulate the unsaturation levels of PS^42^.

## Conclusions

The data herein represent a subset of a larger lipidomic evaluation of chicken hepatic tissue. The results confirmed that FG strain, such as the Ross 308 genotype, showed a lipid metabolism connected with storage and structural roles as demonstrated by TG, PE and PC profiles. Therefore, they use most of the metabolic energy for storage in their tissues and have poor de novo synthesis of LC PUFA, as demonstrated by the FA profile, the lower FADS2 expression and the Δ6d enzyme activity. Conversely, the Leghorn chickens showed a relevant production of n-3 LC PUFA, being most abundant as NEFA (the free ones) and showing a higher unsaturation level in PS, which are the PL most involved in DHA incorporation. The crossbreed seems to have a not clearly defined lipid metabolism.

It could be argued that broilers require increased concentrations of pre-formed LC PUFA in their diet to enrich their muscle content to the proportion observed in Leg and LxR (with the same fat content). Indeed, in the last two strains the more efficient conversion of precursors (ALA and LA) to LC PUFA allows a lower dietary LC PUFA supplementation to achieve a similar degree of enrichment.

Further insights will be investigated on the energy partition in lipid metabolism of chickens.

## Methods

### Animals and housing system

The trial was carried out in the experimental section of the Department of Agricultural, Food and Environmental Science (University of Perugia, Italy) according to EU Regulation 834/07 and Directive 210/63/EU and transposed as D.L. 26/2014 art. 25 for experimental animal and other scientific purposes. The animals were reared in the experimental farm of the University of Perugia, following an experimental protocol approved by the University Ethics Committee (ID: 62,700). The animals were slaughtered in the commercial slaughterhouse and the tissues of interest were recovered from the dead animals as reported below.

The three chicken genotypes, Leghorn (Leg) as SG, Ross 308 (Ross) as FG and their crossbreed Leghorn x Ross 308 (LxR, produced by mating SG hens with FG cocks), were reared indoors at the same density (5 chickens/m^2^) and fed ad libitum with the same standard diet, which was formulated according to the nutritional recommendations for broiler chickens. The diet was divided into three periods: starter 0–21 d, grower 22–60 d and finisher 61–81 d (Supplemental material table S2). The Leghorn is a local breed and it was selected and conserved in the Department for more than sixty years^43,44^. For each genotype, a total of 30 animals were reared and divided into 3 replicates of 10 birds each. At 81 d of age (minimum age for SG strain), all birds were slaughtered in the same slaughterhouse approved by the EU regulation. The slaughtering procedure provided stunning by electronarcosis, bleeding by incision of the major blood vessels of the neck, removal of the feathers and hanging of the carcass.

### Sample collection

For each genotype, a total of 6 carcasses (2 carcasses/replicate) were sampled, and the liver and *Pectoralis major* muscle (breast) were collected. The liver was promptly removed and thoroughly washed with saline solution. Within a few minutes, tissue samples were cut into appropriately sized pieces, as following reported. Approximately 1 g was imbibed in 5 volumes RNAlater (Sigma, Milan, Italy) as indicated by the manufacturer for mRNA expression quantification (after 1 day at + 4 °C RNAlater was removed and samples placed -80 °C until analysis). About 10 g was removed and stored at -20 °C for enzyme activity evaluations (5 g) and lipid profile (to proceed with freeze-drying). Approximately 10 g of frozen samples was freeze-dried (Edwards freeze drying, Milan, Italy) for 24 h under vacuum (50 mTorr). Each sample was weighed to determine the water loss. After drying, the samples were carefully mechanically minced and stored at − 80 °C prior to analysis of the lipid composition (1 week later).

The *Pectoralis major* muscle was also removed (about 50 g) and stored at − 20 °C for FA quantification.

### Preparation of total RNA, cDNA synthesis, FADS2 cloning and sequencing

The preparation of total RNA, cDNA synthesis, FADS2 cloning and sequencing were performed as reported in Castellini et al.^45^ Total RNA was extracted from chicken liver using PureYield RNA (Promega, Italy), following the manufacturer’s protocol (#TM279). The quantity of the extracted RNA was calculated spectrophotometrically by using the absorbance at 260 nm (NanoDrop, Thermo Scientific, Italy), whereas the integrity of RNA was assessed by agarose gel electrophoresis. Crisp 18S and 28S bands, detected by ethidium bromide staining were indicator of the intact RNA. After extraction, total RNA was reverse transcribed into cDNA in a mix containing oligo dT16 primer, and dNTPs. This mix was heated, chilled on ice, and then reverse transcription buffer, DTT, RNaseOUT, and Moloney murine Leukaemia virus (M-MLV) reverse transcriptase were added, as described in the M-MLV Reverse Transcriptase kit (Invitrogen). To perform PCR, an aliquot of the resulting cDNA was amplified with GoTaq Polymerase (Promega) in a final volume containing buffer, dNTPs, and the primer set (FADS2 sense + antisense) designed by us (Supplemental material Table S3). To design the primers, we firstly performed a BlastN search (http://www.ncbi.nml.nih.gov/BLAST/) at the GeneBank database for FADS2 gene in *Gallus gallus*, finding a cDNA sequence with the accession number: NM_001160428.2. This sequence was used to design the primers FADS2-sense1 + antisense1 (Supplemental material Table S3; Supplemental material Figure S1). The PCR amplifications for FADS2 were performed using an automated Thermal Cycler (Mycycler, Biorad). An aliquot of each PCR reaction was then electrophoresed on agarose gel and bands were detected by ethidium bromide staining. The PCR product from primer amplification were then cloned using the pGEMT-Easy cloning vector system (Promega, Italy) and subsequently sequenced in both directions (T7 and SP6).

### Quantitative real-time RT-PCR

The number of gene transcript copies of FADS2 gene was quantified by comparing them with a standard graph constructed using the known copy number of mRNAs of this gene^45^. For this, a forward (FADS2_T3promoter) and a reverse (FADS2_antisense2) primer were designed based on the cDNA sequence of *Gallus gallus* (acc.nr. NM_001160428.2) (Supplemental material Table S3; Supplemental material Figure S1). The forward primer was engineered to contain a T3 phage polymerase promoter gene sequence at its 5′ end (Supplemental material Table S3) and used together with the reverse primer in a conventional RT-PCR of total chicken liver RNA. RT-PCR products were then evaluated on a 2.5% agarose gel stained with ethidium bromide, cloned using pGEM®-T cloning vector system (Promega, Italy), and subsequently sequenced. This primer pair was used to create templates for the in vitro transcription of mRNAs. In vitro transcription was performed using T3 RNA polymerase and other reagents supplied in the Promega RiboProbe In Vitro Transcription System kit according to the manufacturer’s protocol. The molecular weight (MW) of the in vitro-transcribed RNA for each gene was calculated according to the following formula:

$${\text{MW}} = \left( {{\text{no of A bases}} \times {329.2}} \right) + \left( {{\text{no of U bases}} \times {306.2}} \right) + \left( {{\text{no of C bases}} \times {305.2}} \right) + \left( {{\text{no of G bases}} \times {345.2}} \right) + {159}.$$

The mRNAs produced by in vitro transcription were then used as quantitative standards in the analysis of experimental samples using one-step TaqMan EZ RT-PCR Core Reagents (Life technologies, Italy). RT- PCR conditions were: 2 min at 50 °C, 30 min at 60 °C, and 5 min at 95 °C, followed by 40 cycles consisting of 20 s at 92 °C, 1 min at 62 °C. The Ct values obtained by amplification were used to create standard curves for target genes.

### Quantitation of FADS2 transcript by One-step Taqman RT-PCR

One hundred nanograms of total RNA extracted from the experimental samples was subjected, in parallel to tenfold-diluted, defined amounts of standard mRNAs, to real-time PCR under the same experimental conditions as those used for the establishment of the standard. Real-time Assays-by-DesignSM PCR primers and Taqman gene-specific fluorogenic probes were designed by Life Technologies (LT). The sequences of primers (FADS2 sense3 + antisense 3) and of the Taqman probe are presented in Supplemental material Table S3. One Step TaqMan® PCR was performed on a StepOne Real Time PCR System (LT, Italy). Data from Taqman® PCR runs were collected with StepOne Sequence Detector Program. The reaction efficiency was in the range 90–92%. Furthermore, a minus-reverse transcriptase control (“No Amplification Control” or NAC) was included in qRT-PCR experiments. The NAC was a mock reverse transcription containing all the RT-PCR reagents, except the reverse transcriptase. No product was seen in the NAC, which indicates that contaminating DNA was not present in the sample^45^.

### Quantitation of Δ6-Desaturase activities

Microsomes were isolated from fresh chicken liver as reported in Castellini et al.^45^ The Δ6-desaturase enzymatic activity was estimated by measuring the amount of [1-14C]18:3 n-6 (γ-linolenic) produced from [1-14C]18:2 n-6 (LA) (Perkin Elmer) in buffer A. 0.5 mL assay mixture contained the following concentrations of cofactors and reagents: 4 mM ATP, 0.1 mM CoA, 1.25 mM NADH, 0.5 mM nicotinamide, 5 mM MgCl2, 62.5 mM NaF, 1.5 mM GSH and 35 of unlabeled fatty acid nmol of substrate. For each sample, about 3 nmol [1-14C]18:2 n-6 were blended with 30 nmol of unlabeled fatty acid and resuspended in buffer A complexed with 0.02% bovine serum albumin (free fatty acid). The specific radioactivity of substrate (~ 4 nCi/nmol) was calculated by liquid scintillation counter. The reaction was started by addition of 2 mg of microsomal proteins and assay mixture was incubated in shaking water bath at 37 °C for 30 min. Under these assay conditions the rate of desaturation of LA was linear with respect to the microsomal protein, substrate concentration and incubation time. After stopping the reaction by 10% KOH in methanol, total lipids were saponified by heating the methanolic KOH mixture for 60 min at 80 °C. The mixture was then acidified with 8 M HCl (pH 1–2) and fatty acid were extracted with hexane in a 3 steps extraction. The fatty acid was then converted to methyl ester by heating for 2 min at 100 °C with 0.5 ml of 14% (w/w) BF3-methanol, and extracted with hexane in a 3 steps extraction. The purified fatty acid methyl esters (FAME) were dissolved in hexane with butylated hydroxytoluene as an antioxidant, flushed with N_2_, and kept in a -20 °C freezer until analysis. The distribution of radioactivity between the 18:2 n-6 substrate and the 18:3 n-6 product of Δ6-desaturase activity was determined by thin-layer chromatography (TLC) with silica gel plates impregnated with 10% (w/w) AgNO3 (SiliCycle, Canada). Standard methyl esters of 18:2 n-6 and 18:3 n-6 were spotted near the labelled FAME to identify reaction product. Plates were developed in hexane/diethyl ether (2/3, v/v) for the separation of trienes from tetraenes. The spots were made visible under UV light by spraying with 2’,7’-dichlorofluorescein [0.2% (w/v) in ethanol]. Radioactive spots were also identified by Istant Imager (Packard) and scraped off directly into the scintillation vials and counted for radioactivity with 4 mL of scintillation flour by using a liquid scintillation analyzer (Tri-carb Packard, model 1600 CA).

### Untargeted lipidomic analysis of liver

Approximately 0.2 g of each sample was extracted with 2 ml of a methanol:MTBE:chloroform (MMC) mixture (40/30/30, v/v/v) containing 1 mg/l 2,6-di-*t*-butyl-*p*-hydroxytoluene (BHT) as an antioxidant (modified by Pellegrino et al.^46^). The samples were then vortexed well and shaken at room temperature for 30 min (950 rpm). Then, the samples were centrifuged for 10 min at 8,000 rpm. The supernatants were collected in fresh tubes, and 2 μl was used for LC/MS analysis. The LC/MS system consisted of a Dionex UltiMate 3000 series (Thermo Fisher Scientific, Waltham, MA, USA) with a binary pump, a thermostated autosampler and a column compartment coupled with a Thermo Q-exactive mass spectrometer (Thermo Fisher Scientific, Waltham, MA, USA). Liquid chromatography separation was performed at 45 °C using a Kinetex F5 reverse-phase column (Phenomenex Inc., Bologna, Italy) at the flow rate of 0.65 ml/min. The mobile phases consisted of 5 mM ammonium formate and 0.1% formic acid in water (solvent A) and 5 mM ammonium formate and 0.1% formic acid in isopropanol (solvent B). A gradient elution was used for lipid separation as follows: time 0 min, solvent A 80%, solvent B 20%; time 3 min, solvent A 60%, solvent B 40%; time 16 min, solvent A 40%, solvent B 60%; time 16.5 min, solvent A 30%, solvent B 70%; time 24 min, solvent A 26%, solvent B 74%; time 28 min, solvent A 5%, solvent B 95%; and time 30, stop run. All solvents were purchased from Sigma-Aldrich.

Mass spectrometry analysis was performed as a first step in the positive/negative ion switching mode within full MS scan mode. The alpha and gamma linolenic acid was differently identified based on the proportion reported in previously published data^47^. Then, the high-resolution mass list obtained was processed with Lipostar software (version 1.3.0, Molecular Discovery Ltd., UK) to perform preidentification (by mass searching within a library of approximately 800,000 in silico fragmented lipids) of potential lipid species on the basis of the mass and retention time values. The masses of interest generated after this preidentification step were used for inclusion lists applied to reanalyze in DDS mode a reduced number of samples automatically selected by Lipostar to obtain MS/MS data^48^. The MS/MS data were then imported into the data matrix generated by Lipostar to perform the final lipid identification step based on exact mass, retention time and MS/MS fragmentation. Automatically generated data were visually inspected, and only high-confidence data were selected for the statistical analysis. The confidence of results was automatically evaluated by Lipostar, only compounds with characteristic fragments were included for statistical evaluations.

### Fatty acid profile evaluation of breast muscle

Total lipids were extracted and quantified from the muscle (10 g), accurately minced by an ultraturrax (IKA T18, Steroglass, Italy) following the method reported by Folch et al.^49^ To obtain fatty acid methyl esters, the lipid extract was dried with a rotary evaporator (Strike 10 Steroglass, Italy), and 1 ml of *n*-hexane was added. Finally, the trans-methylation procedure was performed with 0.5 ml of 2 M KOH–methanol solution at 60 °C for 15 min. To calculate the amount of each FA, heneicosanoic acid was used as the internal standard (C21:0, Sigma-Aldrich analytical standard), and data were expressed as mg/100 g of meat (quantitative evaluation) and % of total FA (qualitative evaluation). The average amount of each FA was used to calculate the sum of total PUFA and LC PUFA of the n-3 and n-6 series and the proportion of each LC PUFA respect to the total LC PUFA. Also the muscle enzyme activity was estimated as products/precursors ratio for both series.

### Statistical evaluation

The NEFA, TG and PL of the liver, FADS2 gene expression, Δ6d activity and FA profile of breast muscle were statistically analyzed using one-way ANOVA (SPSS, Statistics for Data Analysis v. 27) with the fixed effect of the genotype. Statistical significance was set at 0.05, and differences were assessed using post hoc Tukey’s test. Variability in the data is shown as the standard error for liver parameters and root mean square error (RMSE) for muscle parameters.

## Supplementary Information

Supplementary Information.

## Abbreviations

PUFA: Polyunsaturated fatty acids;
FA: Fatty acids
LC PUFA: Long chain polyunsaturated fatty acids
ALA: α-Linolenic acid
LA: Linolenic acid
EPA: Eicosapentaenoic
DPA: Docosapentaenoic
DHA: Docosahexaenoic
ARA: Arachidonic acid
AdA: Adrenic acid
Δ6d: Δ-6 Desaturases
TG: Triglycerides
PL: Phospholipids
VLDL: Very-low-density lipoprotein particles
NEFA: Non-esterified fatty acids
SG: Slow-growing genotype
FG: Fast-growing genotype
MG: Medium-growing genotype
CP: Crude protein
ME: Metabolizable energy
MMC: Methanol:MTBE:Chloroform
BHT: 2,6-Di-t-butyl-p-hydroxytoluene
RMSE: Root Mean Square Error
PE: Phosphatidylethanolamine
PC: Phosphatidylcholine
PS: Phosphatidylserine
UNS: Unsaturations
LPL: Lipolytic enzyme
FADS2: Fatty Acids 2 gene
