# Supplementary material for: Lipid metabolism analysis in liver of different chicken genotypes and impact on nutritionally relevant polyunsaturated fatty acids of meat
Source: Sci Rep. 2022 Feb 3;12:1888. doi: 10.1038/s41598-022-05986-2 (PMC8814176; doi:10.1038/s41598-022-05986-2)
Supplement: Supplementary file 1 — Supplementary Information. [file 41598_2022_5986_MOESM1_ESM.doc]

Table S1. Estimated composition in polyunsaturated fatty acids (PUFA) of phospholipids (PL) classes

| PE/PC/PS^1^ | Estimated Number of PUFA included | LC PUFA^2^ n-3 included |
| --- | --- | --- |
| 2 UNS | n:1,n:1-PL | none |
| 3 UNS | n:1,n:2-PL | none |
| 4UNS | n:1,n:3-PL  n:2,n:2-PL | none |
| 5 UNS | n:1,n:4-PL  n:2,n:3-PL | none |
| 6 UNS | n:1,n:5-PL  n:2,n:4-PL  n:3,n:3-PL | 1 |
| 7 UNS | n:1,n:6-PL  n:2,n:5-PL  n:3,n:4-PL | 1 or more |
| ≥8 UNS | n:2,n:6-PL (8 UNS)  n:3,n:5-PL(8 UNS)  n:3,n:6-PL (9 UNS)  n:4,n:6-PL (10UNS)  n:5,n:5-PL (10 UNS)  n:5,n:6-PL (11 UNS)  n:6,n:6-PL (12 UNS) | 1 or more |

^1^ Phosphatidylethanolamine: PE; Phosphatidylcholine: PC; Phosphatidylserine: PS; UNS: unsaturation level. ^2^ LC PUFA: Long Chain Polyunsaturated Fatty Acids.

Table S2. Formulation, chemical analysis and fatty acids profile of chicken feed

|  |  | **Starter** | **Grower** | **Finisher** |
| --- | --- | --- | --- | --- |
| **Ingredients** |  |  |  |  |
| Corn | % | 53.92 | 55.95 | 53.11 |
| soybean meal 48% | % | 30.23 | 24.67 | 15.69 |
| Extruded corn flour | % | 5.08 | 8.90 | 11.45 |
| wheat | % | 5.00 | 5.00 | 15.00 |
| Dicalcium phosphate | % | 1.71 | 1.58 | 1.21 |
| Calcium carbonate | % | 1.23 | 1.16 | 1.29 |
| Corn gluten 70 | % | 1.00 | 1.00 | / |
| Soybean oil | % | 0.62 | 0.54 | 1.15 |
| Vitamin supplement | % | 0.40 | 0.40 | 0.40 |
| salt | % | 0.20 | 0.18 | 0.23 |
| Mineral supplement | % | 0.16 | 0.16 | 0.11 |
| Sodium bicarbonate | % | 0.15 | 0.15 | 0.15 |
| **Chemical composition** |  |  |  |  |
| Dry Matter (DM) | % | 87.80 | 87.89 | 88 |
| Crude protein | % DM | 24.01 | 22.16 | 18.41 |
| Lipids | % DM | 3.99 | 3.98 | 4.55 |
| Crude fiber | % DM | 3.48 | 3.58 | 3.60 |
| Ashes | % DM | 6.92 | 6.43 | 5.78 |
| Metabolizable energy | kcal/kg | 3245 | 3242 | 3295 |
| Vitamin A | U.I. | 11385 | 11377 | 11364 |
| Vitamin E | Mg | 36.43 | 36.41 | 36.37 |
| **Fatty acids profile** |  |  |  |  |
| 14:0 | % of total FA | 1.05 | 1.04 | 1.10 |
| 16:0 | “ | 13.98 | 13.9 | 13.11 |
| 18:0 | “ | 4.1 | 4.2 | 4.32 |
| 16:1 | “ | 1.02 | 1.02 | 0.90 |
| 18:1 | “ | 25.03 | 24.9 | 24.93 |
| 18:2n-6, LA | “ | 48.2 | 48.2 | 48.81 |
| 18:3n-3, ALA | “ | 6.62 | 6.74 | 6.83 |

Table S3. Sequences of the primers used in the work

| Primer | Sequence 5’ – 3’ | Purpose |
| --- | --- | --- |
| FADS2-sense1 | AGTACGGCAAGAAGAAGCTGA | RT-PCR |
| FADS2-antisense1 | CACCACCTGTTCCCAACAATG | RT-PCR |
| FADS2_T3promoter | *caattaaccctcactaaaggga*CGGCAAGAAGAAGCTGA | mRNA std.curve |
| FADS2_antisense2 | CTGGAGAGCCACTGGTTTGT | mRNA std.curve |
| FADS2 – sense 3 | CCCGTGTATTTCCAAATCCAAATCAT | Real-time |
| FADS2 – antisense 3 | AGCTACTACATGCGCTATTTCA | Real-time |
| Taqman® FADS2 | GTTCTGGGCGGACCTGG | Real-time |

Figure S1. *Gallus gallus* fatty acid desaturase 2 (FADS2), mRNA

NCBI Reference Sequence: NM_001160428.2

>gi|261878589:149-1483 Gallus gallus fatty acid desaturase 2 (FADS2), mRNA

ATGGGGAAGGGGGGCGAGAAAGGAGAGGAGTCCGGGGAGTGCAAGCCGCAGGTCCGCTCCTACACCTGGGAGGAGATCCAGAAGCACAACCTGAGGACGGACAGGTGGCTGGTGATAGAGCGGAAGGTTTACAATGTCACCCAGTGGGCGAGCAGGCACCCGGGCGGCCAGCGGGTCATCGGCCACTGCGCCGGCGAGGATGCCACGGATGCATTCCAGGCCTTCCACATCAATCCCAGCTTGGTGCAGAAGTTTCTCAAGCCATTACTTATTGGAGAGCTTGCTCCAGGGGAGCCCAGCCAGGACCGAGATAAAAATTCCCAGCTGGTGGAGGATTTTCGGACCCTGAGGAAGACAGCAGAGGACATGAACTTATTCAGAGCCAGTCCTTTGTTCTTCTCTCTTTACTTGGCCCATATCATTGCAATGGAAGCATTGGCTTGGCTAATGGTTTCATACTTCGGTACCGGCTGGATCACAACTCTAATCCTTGCCTGCATCCTTGCAACTTCCCAGGCCCAGGCAGGTTGGCTGCAACATGACTTTGGACACCTCTCTGTCTTTAAGAAGTCTTCCTGGAACCACATCGTCCACAAGTTTGTGATTGGACACCTTAAGGGTGCCTCTGCAAACTGGTGGAACCATCGTCACTTCCAACATCACGCTAAGCCCAACATATTCAAGAAAGACCCAGATGTGAACATGCTGCATATTTTTGTCCTTGGCGAAAGTCAGCCTATTGAGTACGGCAAGAAGAAGCTGAAGTACCTGCCTTACAACCACCAGCATGAGTACTTCTTCCTCATCTTCCCACCTCTGCTCATCCCCGTGTATTTCCAAATCCAAATCATCTCAACCATGATCAAGCGCAGGTTCTGGGCGGACCTGGCCTGGGCCATCAGCTACTACATGCGCTATTTCATCACATACATCCCATTCTATGGCATTCTGGGATCCCTGTTTCTCCTCACTTTTGTCAGGTTTCTGGAGAGCCACTGGTTTGTATGGGTCACTCAGATGAATCACATTCCAATGGAAATTGATTGTGAGAAGCACAAAGACTGGCTTAGCTCTCAGCTGGCAGCCACCTGCAATATTGAGCAATCCTTTTTCAATGACTGGTTCACCGGGCACCTGAACTTTCAAATTGAGCACCACCTGTTCCCAACAATGCCACGGCACAATTTCTGGAAAATCAAACCCTTGGTGAAGTCATTATGTGCCAAGTATGGAGTGCATTACGAAGAGAAGTCTCTTGGAAAAGCATTTGTAGACATAGTTGGGTCACTAAAGAAATCTGGAGATTTATGGCTGGATGCTTACCTCCACAAGTGA
